# A Larger Social Network Enhances Novel Object Location Memory and Reduces Hippocampal Microgliosis in Aged Mice

**DOI:** 10.3389/fnagi.2018.00142

**Published:** 2018-05-31

**Authors:** Bryon M. Smith, Xinyue Yao, Kelly S. Chen, Elizabeth D. Kirby

**Affiliations:** ^1^Department of Psychology, The Ohio State University, Columbus, OH, United States; ^2^Department of Neurology and Neurological Sciences, Stanford University, Stanford, CA, United States; ^3^Department of Neuroscience, The Ohio State University, Columbus, OH, United States; ^4^Center for Chronic Brain Injury, The Ohio State University, Columbus, OH, United States

**Keywords:** hippocampus, social, aging, neuroinflammation, environmental enrichment, neurogenesis, microglia

## Abstract

The mammalian hippocampus shows marked decline in function with aging across many species, including humans and laboratory rodent models. This decline frequently manifests in memory impairments that occur even in the absence of dementia pathology. In humans, a number of factors correlate with preserved hippocampal memory in aging, such as exercise, cognitive stimulation and number of social ties. While interventional studies and animal models clearly indicate that exercise and cognitive stimulation lead to hippocampal preservation, there is relatively little research on whether a decline in social ties leads to cognitive decline or vice versa. Even in animal studies of environmental enrichment in aging, the focus typically falls on physical enrichment such as a rotating cast of toys, rather than the role of social interactions. The present studies investigated the hypothesis that a greater number of social ties in aging mice would lead to improved hippocampal function. Aged, female C57/Bl6 mice were housed for 3 months in pairs or large groups (7 mice per cage). Group-housed mice showed greater novel object location memory and stronger preference for a spatial navigation strategy in the Barnes maze, though no difference in escape latency, compared to pair-housed mice. Group-housed mice did not differ from pair-housed mice in basal corticosterone levels or adult hippocampal neurogenesis. Group-housed mice did, however, show reduced numbers of Iba1/CD68+ microglia in the hippocampus. These findings suggest that group housing led to better memory function and reduced markers of neuroinflammation in aged mice. More broadly, they support a causative link between social ties and hippocampal function, suggesting that merely having a larger social network can positively influence the aging brain. Future research should address the molecular mechanisms by which a greater number of social ties alters hippocampal function.

## Introduction

Aging is associated with a prominent decline in cognitive function (Bettio et al., 2017). In humans, this decline can limit quality of life and restrict independance. The memory functions mediated primarily by the hippocampus are particularly vulnerable to decline with age (Bartsch and Wulff, 2015; Bettio et al., 2017). Age-related decline in hippocampal memory occurs even absent more obvious pathological conditions, such as Alzheimer’s disease, and is similarly found in other mammals, including non-human primates and rodents (Leal and Yassa, 2015).

As the average human lifespan increases, the economic and social costs of age-related cognitive decline are growing (Ballesteros et al., 2015; Sanderson et al., 2017). One approach to developing treatments for cognitive decline has been to pursue individual behavioral factors that are associated with preserved function in old age. For example, a life history of exercise and cognitive enrichment correlates with protection from cognitive decline in human aging and there is some evidence that introducing exercise and enrichment in old age can provide protection from further decline (Ballesteros et al., 2015; Bettio et al., 2017). Studies in rats and mice have similarly shown benefits of exercise and environmental enrichment for hippocampus-dependent memory function in aged animals (van Praag et al., 2000; Lee et al., 2012).

A larger social network is also a well-established predictor of protection from cognitive decline in humans, but it has received much less attention as a potential treatment. Aged individuals with more social ties show less cognitive decline than those with fewer ties in both longitudinal and cross-sectional studies (Bassuk et al., 1999; Seeman et al., 2001; Béland et al., 2005; Ertel et al., 2008; Haslam et al., 2015). This association between greater social ties and preserved cognition even extends to pathological conditions such as Alzheimer’s disease (Fratiglioni et al., 2004). However, aging is generally associated with reduced social interactions (Shaw et al., 2007; Bettio et al., 2017). This age-related contraction of social network can be found in non-human primate species, as well (Almeling et al., 2016; Crockford, 2016), suggesting that the decrease in social interaction with increasing age may have conserved biological underpinnings. This potentially self-driven reduction in social interactions combined with the correlative nature of currently available data make it unclear whether a large social network protects the brain from impairment or whether a loss of mental acuity drives withdrawal from social relationships. In addition, social connectedness is difficult to control in humans and effective study of social support as an intervention variable has proved challenging (Hogan et al., 2002).

Remarkably, very few animal studies of age-related cognitive decline to date have investigated the social network as a neuroprotective factor. Those studies that do address social interaction effects on cognition often combine it with other forms of physical enrichment (larger cages, toys), making the role of social interaction difficult to isolate (van Praag et al., 2000; Lee et al., 2012). Studies that more specifically investigate social interactions primarily compare socially isolated animals to those that are group housed (Speisman et al., 2013). Complete social isolation is a severe stressor in rodents (and humans) and, though humans show reductions in social network size with aging, complete isolation is not commonly found (Shaw et al., 2007). It therefore remains unclear from both human and rodent research whether larger social networks alone can contribute to preserved hippocampal function in aged individuals.

We hypothesized that having a larger social network would improve hippocampal function in aging mice. Using wild-type, aged female mice, we investigated the effect of living in pairs (2 mice) or large groups (7 mice) on hippocampus-dependent memory function and on histological markers of age-related hippocampal dysfunction.

## Materials and Methods

### Animals

Female C57/Bl6J mice aged 15 months were housed 4/cage upon arrival in standard ventilated cages for 1 week before experimental procedures began. Mice had *ad libitum* access to food and water throughout all experiments and were maintained on a 12 h light cycle with lights on at 6:30 h. Behavioral testing was performed between 6:30 h and 14:30 h. This study was carried out in accordance with the recommendations of the National Institutes of Health Guide for the Care and Use of Laboratory Animals and the Veterans Administration Palo Alto Committee on Animal Research. The protocol was approved by the Veterans Administration Palo Alto Committee on Animal Research.

### Novel Object Location Pre-testing

Pre-testing in the hippocampus-dependent novel object location task was used to assure similar baseline memory performance across groups. After 1 week acclimation to housing, mice were handled each day for 3 days. On day 4, mice were moved in their home cage to a behavioral testing room and allowed to acclimate for 30 min. Mice were placed in an open field (45 × 45 × 45 cm) made of opaque plastic for 6 min. After a 1.5 h inter-trial interval (ITI), two distinct objects were placed in the open field and mice were given three 6-min trials to explore those objects with a 1.5 h ITI. Mice were returned to their home cages in the testing room between trials. At the end of training, mice were returned to the colony room. 24 h after training, mice were again acclimated to the testing room for 30 min. The same two objects from training were placed in the open field with one object in the same position as during training and the other placed in a new location, equidistant from the open field walls as the unmoved object. Mice were allowed to explore these objects in the open field for 6 min. After testing, mice were returned to their home cages then returned to the colony room.

The open field and objects were cleaned with 70% ethanol between trials and between mice to reduce odor cues. Objects were secured to the open field floor with tape to assure that mice could not move the objects. Behavior was recorded by video and TopScan software (CleverSys Inc.). Time spent in the center 70% of the open field and speed of travel were quantified by TopScan. Object investigation was scored by a blinded observer. Investigation was defined as a mouse with nose less than 1 cm away from and pointed at an object.

### Group Assignments

Mice were assigned to housing conditions to counterbalance: (1) performance on the novel object location pre-testing; and (2) familiarity of mice from the initial week of housing. Mice were assigned to live either 2/cage (*n* = 10, 5 cages, “pairs”) or 7/cage (*n* = 14, 2 cages, “groups”). Two mice in the pair-housed condition were euthanized for overgrooming 3 weeks before behavioral testing and their cagemates were removed from further analysis, resulting in a final *n* = 6 for pair-housed mice. Mice living in pairs were all unfamiliar and had not been previously housed together since arrival. Mice living in groups had at most two mice who had been housed together since arrival. Percent time investigating the novel object location in pre-testing was similar between housing conditions: 56.931 ± 9.10% (SEM) pair-housed vs. 58.65 ± 6.08% (SEM) group-housed mice; *t* = 0.21, *p* = 0.83.

Experimental housing began 1 day after novel object location pre-testing. Mice were housed with new cagemates in a clean, large mouse cage (28 w × 28 l × 12.7 h cm) with corn-cob bedding. Mice were provided with one nestlet for every two mice in the cage throughout the experiment. Mice were weighed and cages were changed weekly. These housing arrangements were maintained for 3 months.

### Fecal Matter Sampling

Fecal matter was collected from each cage every 2 weeks after experimental housing began. Twenty-four hours after changing to a clean cage, fecal pellets were collected in 1.5 ml tubes using forceps. Samples were stored at −20°C until assayed for corticosterone.

### Bromodeoxyuridine Injections

After 2 months in experimental housing groups, all mice were injected with 150 mg/kg bromodeoxyuridine (BrdU, Sigma) dissolved in sterile saline once per day for 4 days.

### Novel Object Location and Recognition Post-testing

After 3 months in pair or group housing conditions, mice were handled each day for 3 days. On the 4th day, they were transferred to individual holding cages with food and water, and allowed to acclimate to the behavior testing room for 45 min. Mice were given three 10-min trials with a 5 min ITI in the empty open field used for pre-testing. Mice were then returned to their home cages in the colony room. The next day, mice were allowed to acclimate to the behavior testing room for 45 min in the same individual holding cages used the previous day. Two, distinct novel objects were placed in the open field and mice were allowed to explore for 10 min. After a 5 min ITI in the holding cage, mice were returned to the open field for 10 min with the same two objects but one was placed in a new location equidistant from the walls as the other, un-moved object (novel object location task, NOL). After a 5 min ITI, the object that did not change location in the previous trial was replaced by a new object the mice had never encountered before and mice were allowed another 10 min of exploration (novel object recognition task, NOR). Three objects total were used and they were counterbalanced across mice. Half of the mice saw objects 1 and 2 paired during training and NOL, and objects 2 and 3 in the NOR task. The other half of the mice saw objects 2 and 3 paired together during training and NOL, and objects 1 and 2 in the NOR task. Object 2 was always the object that moved and object 1 or 3 was the novel object, depending on which one was used in the training and NOL trials. This counterbalancing allowed comparison of investigation of object 1 vs. 2 and 2 vs. 3 during training to assure that mice on average showed no strong inherent preference for investigation of one object over the other. In the training trial, percent time investigating object 2 over object 1 was 48.39 ± 2.92% (SEM, *n* = 11) and percent time investigating object 2 over object 3 was 47.14 ± 2.34% (SEM, *n* = 9). Neither of these percent preferences was significantly different from 50% by one sample *t*-test (object 2 vs. 1: *t* = 0.553, *p* = 0.592; object 2 vs. 3: *t* = 1.22, *p* = 0.257). Mice were handled and placed in the open field by an experimenter blind to housing condition. After the final trial, mice were returned to their home cages in the colony room. Cleaning and behavioral quantification was performed as in pre-testing.

### Barnes Maze Testing

One day after novel object testing, mice began training in the Barnes maze. The Barnes maze is a hippocampus-dependent task and the version used here has been previously shown to be sensitive to rejuvenation of memory function in aged C57/Bl6 mice (Castellano et al., 2017). The Barnes maze consisted of a flat, circular platform with 40 holes. The platform was elevated 40 cm from the floor and an escape tube made of similar material as the platform was affixed beneath one hole. Mice received 4 days of Barnes maze training with four trials per day. Each day, mice were transferred to their individual holding cages and transported to the behavioral testing room. Mice were placed on the maze, near the outer edge, under an opaque box for 10 s before being released to explore the maze. If a mouse failed to enter the escape hole after 90 s, it was guided to the hole and into the escape by an experimenter. Throughout the duration of the trial, a 2 kHz tone was played. An overhead light was also illuminated to provide additional motivation to escape the maze. Within each day, the escape was located under the same hole for all four trials and the release sites were rotated for each trial relative to the escape. Between days, the location of the escape hole moved 90° compared to the previous day. Once a mouse entered the escape, the 2 kHz tone was terminated. The mouse was then returned to its holding cage in the behavior testing room. The maze was cleaned with 70% ethanol between trials. Mice were run in groups of four, yielding an ITI of 5–10 min. After four trials, mice were returned to their home cages in the colony room. All trials were video recorded from a ceiling mounted digital camera. Primary escape latency (the latency to find the escape) and search strategy were scored by a blinded observer for each trial (Harrison et al., 2006; O’Leary and Brown, 2013). Search strategy was categorized as serial if a mouse searched three or more consecutive peripheral holes before finding the escape. Searching non-consecutive holes or navigating directly to the escape was categorized as a spatial search strategy.

### Sociability and Social Memory Testing

One day after the last Barnes maze trial, mice were returned to the behavior testing room in individual holding cages and allowed to acclimate for 30 min. Mice were then placed in a sociability testing apparatus consisting of three chambers (20 l × 40.5 w × 22 h cm each) separated by clear plastic walls with a doorway to connect the center chamber to each side chamber (Moy et al., 2004). In the two outer chambers, wire cages were placed such that a mouse had to enter the chamber to investigate the cage. Mice first were allowed to explore the sociability chambers with the wire cages empty for 5 min. Mice were then confined to the center chamber and a novel object was placed in one wire cage while an unfamiliar, age-matched female mouse was placed in the other wire cage. Experimental mice were then allowed to explore freely for 10 min (social preference task) before being confined to the center again. The novel object was then replaced with a second, unfamiliar age-matched female mouse and experimental mice were allowed to explore freely for 10 min (social memory task). After testing, mice were returned to their home cages in the colony room. The apparatus and the cages were cleaned with 70% ethanol between experimental mice. All behavior was recorded on ceiling mounted video and amount of time in each chamber was scored by a blinded observer. Stimulus mice were age-matched C57/Bl6J female mice that the experimental mice had never encountered before. Stimulus mice were habituated to the sociability apparatus and the wire cages for 5 min/day for 3 days before sociability testing. A total of 14 stimulus mice were used. Stimulus mice were rotated such that they were not used in two consecutive trials.

### Euthanasia and Tissue Collection

Two days after sociability testing, mice received an overdose of Avertin anesthetic. Atrial blood was collected with EDTA followed by transcardial perfusion with ice cold, sterile 0.1 M phosphate buffered saline (PBS). Blood was centrifuged 1000 *g* at 4°C, and plasma was collected and stored at −20°C. After perfusion, brains were harvested and fixed in 4% paraformaldehyde in 0.1 M PB for 24 h at 4°C.

### Corticosterone Assay

Fecal and plasma corticosterone were assayed using a Corticosterone EIA kit (Enzo LifeSciences) as per manufacturer instructions. Plasma was assayed at a 1:50 dilution in assay buffer. Fecal samples were homogenized using mortar and pestle then dried at 37°C for 30 min. Approximately 0.1 g of fecal material from each sample was incubated in 80% methanol with vigorous vortexing for 30 min at room temperature then centrifuged for 10 min at 2500 *g* at room temperature. Supernatant was assayed at 1:10 dilution in assay buffer. Extraction efficiency was measured in four separate samples and was 124.1% ± 30.79 (SD), which was not significantly different from 100% (one-sample *t*-test vs. 100%, *t* = 1.566, *p* = 0.2153). Corticosterone metabolite levels were normalized to the weight of fecal matter extracted.

### Immunohistochemistry

After 24 h fixation, brains were equilibrated in 30% sucrose (wt/vol) in PBS at 4°C then sliced in a series of 1:12 coronal sections at 40 μm on a freezing microtome. Sections were stored at −20°C in cryoprotectant media. Antibody staining was performed using standard procedures on free-floating sections (Kirby et al., 2015; see Table 1). To label cell nuclei, sections were incubated in Hoescht 33342 (1:2000 in PBS, Invitrogen) for 10 min. After 3 × 5 min PBS rinses, all sections were also incubated in 1 mM cupric sulfate in 50 mM ammonium acetate, pH 5.0 for 1 h then rinsed in water to quench lipofuscin autofluorescence. Following 3 × 5 min PBS rinses, sections were mounted on Superfrost Plus microscope glass slides (Fisher Scientific) and protected with Prolong Gold anti-fading medium (Invitrogen). Iba1 and CD68 antibodies have been previously shown to be highly specific to microglial populations (Fulci et al., 2007; Acharya et al., 2016; Qi et al., 2016; Andreou et al., 2017). We further confirmed antibody specificity using brain tissue slices from mice treated with 21 days of PLX5622 diet, which strongly depletes microglial cell populations in the brain (McKim et al., 2017; Supplementary Figure S1). PLX5622 led to an almost complete loss of Iba1 and CD68+ immunoreactivity in the brain.

**Table 1:** Antibodies used with product information.

Antibody	Made in species	Vendor	Product number	Dilution	Antigen retrieval?	Primary or secondary
Iba1	Goat	Abcam	ab5076	1:2000	No	Primary
CD68	Rat	Biorad	MCA1957	1:500	No	Primary
BrdU	Rat	Abcam	OBT0030	1:500	2N HCl at 37°C for 30 min	Primary
DCX	Goat	Santa Cruz Biotechnologies	SC-8066	1:500	No	Primary
NeuN	Mouse	Millipore	MAB377	1:1000	No	Primary
Biotin anti-rat	Donkey	Jackson ImmunoResearch	102649–782	1:500	N/A	Secondary
AlexaFluor647 anti-goat	Donkey	Invitrogen	A21447	1:500	N/A	Secondary
AlexaFluor594 anti-rat	Donkey	Invitrogen	A21209	1:500	N/A	Secondary
Streptavidin AlexaFluor555	N/A	Invitrogen	S21381	1:1000	N/A	Tertiary

### Microscopy and Imaging

Images were acquired on a Zeiss Axio Observer Z1 microscope with Apotome for optical sectioning using a 20× or 40× air objective. Full z-stacks were acquired and merged for analysis. Thresholded area was performed by a blinded observer using ImageJ. Iba1/CD68, DCX and BrdU counts were performed using Zen software (Zeiss) by a blinded observer. Images presented are merged z-stack adjusted only for contrast and brightness.

### Statistical Analysis

All analysis was performed using GraphPad Prism software. Welch’s comparisons were used when comparing pair vs. group housing in a single outcome measure. When pair and group housed mice were quantified repeatedly over time, repeated measures two-way ANOVA (time by group) was used. Repeated measures two-way ANOVA was also used when comparing effects of housing across hippocampal brain regions. *Post hoc* testing was performed using Welch’s comparisons with Bonferroni correction. Welch’s comparisons were chosen because this study had unequal sample sizes and Welch’s test is more robust against Type I error in the case of unequal sample sizes and unequal variances than standard *t*-tests or Sidak’s *post hoc* comparisons (Ruxton, 2006; Derrick and White, 2016). One-sample *t*-tests were used to compare individual group averages to a set standard (such as 50%). *P* < 0.05 was considered significant in all cases.

## Results

### Group Housing Improves Novel Object Location Memory

**Figure 1:**
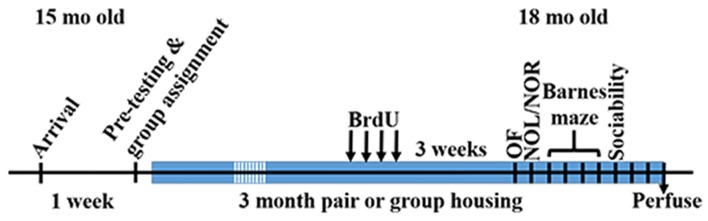
Time of experimental procedures.

**Figure 2:**
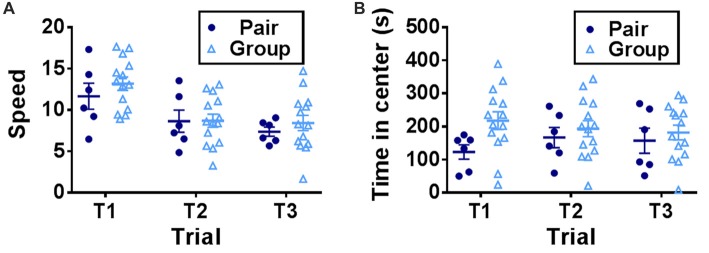
**(A)** Speed of locomotion in the open field decreased over trials but did not differ by housing condition. Two-way RM-ANOVA, main effect of trial (*p* < 0.0001), main effect of housing (*p* = 0.501), trial by housing interaction (*p* = 0.5923), effect of subjects (*p* < 0.0001). **(B)** Time in the center of the open field did not differ by trial or housing. Two-way RM-ANOVA, main effect of trial (*p* = 0.823), main effect of housing (*p* = 0.203), trial by housing interaction (*p* = 0.119), effect of subject (*p* < 0.0001). Each point represents one mouse. Average ± SEM is shown.

To determine the effect of social network size on hippocampal memory in aged mice, 15 month-old female mice were housed in either pairs (2 mice per cage) or groups (7 mice per cage) for 3 months then tested on several behavioral tasks (Figure 1). Mice were first habituated to an open field testing arena over three trials. During these habituation trials, speed of locomotion decreased over the trials but did not differ between pair- and group-housed mice, suggesting similar activity levels between housing conditions (Figure 2A). There was no effect of trial, effect of housing condition or interaction of housing with trial on time in the center of the open field (Figure 2B).

**Figure 3:**
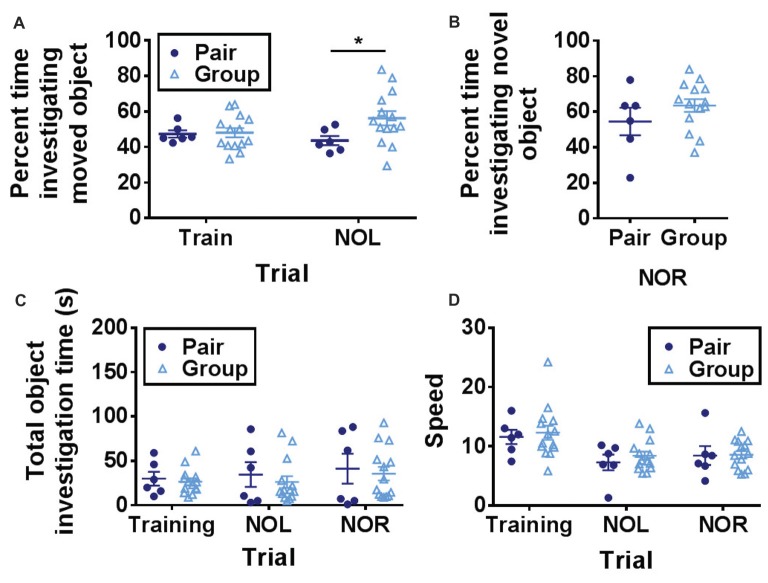
**(A)** Group-housed mice spent significantly more time investigating the moved object in the novel object location task than pair-housed mice. Two-way RM-ANOVA, main effect of trial (*p* = 0.517), main effect of housing (*p* = 0.134), trial by housing interaction (*p* = 0.093), effect of subjects (*p* = 0.172). *p = 0.032 Welch’s test Bonferroni corrected. **(B)** Group-housed and pair-housed mice spent similar percentages of time investigating a novel object. Unpaired Welch’s test, *p* = 0.326. **(C)** Total object investigation time (s) did not differ by trial or housing condition. Two-way RM-ANOVA, main effect of trial (*p* = 0.080), main effect of housing (*p* = 0.625), trial by housing interaction (*p* = 0.862), effect of subjects (*p* < 0.0001). **(D)** Speed decreased over trials but did not differ by housing condition. Two-way RM-ANOVA, main effect of trial (*p* < 0.0001), main effect of housing (*p* = 0.619), trial by housing interaction (*p* = 0.836), effect of subjects (*p* = 0.0004). Each point represents one mouse. Average ± SEM is shown.

One day after open field habituation, mice were placed in the same open field with two novel objects and allowed to explore freely (training trial). After a 1-h ITI, one of the objects was moved to a novel location and preference for investigating the novel object location was measured (NOL trial). NOL is a hippocampus-dependent test of spatial memory in which mice with in-tact memory show a preference for investigating a moved object over a stationary object (Bachevalier and Nemanic, 2007; Komorowski et al., 2009; Barker and Warburton, 2011). This form of memory deteriorates prominently with age (Fahlström et al., 2011). In the NOL trial, group-housed mice spent significantly more time investigating the moved object than pair-housed mice (Figure 3A).

One hour after the NOL trial, the previously stationary object was replaced with a novel object. This novel object recognition (NOR) task is a test of short-term memory that is largely independent of hippocampal function (Bachevalier and Nemanic, 2007; Komorowski et al., 2009; Barker and Warburton, 2011). Consistent with previous findings that aging does not impair object recognition memory (Fahlström et al., 2011), mice showed a strong preference for investigating the novel object over the familiar one (Figure 3B). Percent time spent investigating the novel object did not differ between the two housing conditions (Figure 3B).

Over all three trials (training, NOL and NOR), total object investigation time did not change while locomotor speed decreased and neither was significantly affected by housing condition (Figures 3C,D). These findings suggest that hippocampus-dependent memory function is improved in aged mice housed in large groups compared to mice housed in pairs.

### Group Housing Changes Barnes Maze Search Strategy

**Figure 4:**
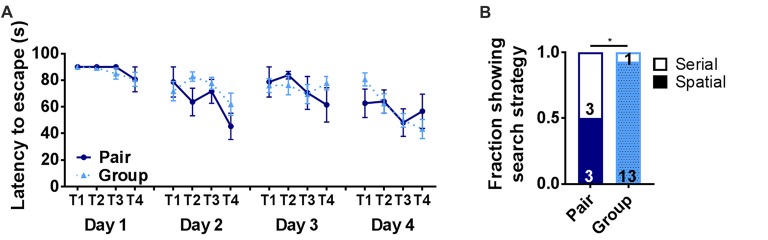
**(A)** Latency to escape in the Barnes maze did not differ on any day of training between pair- and group-housed mice. Day 1: main effect of trial (*p* = 0.131), main effect of housing (*p* = 0.604), trial by housing interaction (*p* = 0.927), effect of subjects (*p* = 0.680). Day 2: main effect of trial (*p* = 0.016), main effect of housing (*p* = 0.224), trial by housing interaction (*p* = 0.308), effect of subjects (*p* = 0.076). Day 3: main effect of trial (*p* = 0.443), main effect of housing (*p* = 0.855), trial by housing interaction (*p* = 0.445), effect of subjects (*p* = 0.080). Day 4: main effect of trial (p =0.012), main effect of housing (*p* = 0.864), trial by housing interaction (*p* = 0.254), effect of subjects (*p* = 0.071). Average ± SEM is shown. **(B)** The number of mice displaying spatial search strategies was significantly higher in grouped over paired housing. *p = 0.028, Chi^2^ test. Fraction of mice is shown with total number in text within bars.

To further assess hippocampus-dependent memory function, pair- and group-housed mice were trained in the Barnes maze. The Barnes maze is a test of hippocampal spatial memory in which mice learn to locate an escape hole on a circular platform. In the present version of the Barnes maze, the escape was in a novel location on each of four testing days (Castellano et al., 2017). Latency to escape did not show any significant effect of housing or trial alone (Figure 4A). To further assess Barnes maze performance, search strategy was determined for each mouse. Several different search strategies have been reported in the Barnes maze, some of which are not dependent on hippocampal memory function (Rosenfeld and Ferguson, 2014). A spatial search strategy, which is the most accurate but most demanding of memory processes, relies on a hippocampal-dependent process of using spatial cues to learn to navigate to the escape. A serial search strategy, in contrast, has also been reported in this task where animals use a hippocampus-independent process of serially searching each hole on the perimeter of the maze until finding the escape. Aged animals are particularly susceptible to relying on a serial search strategy (Bach et al., 1999), which can be rapid but does not typically show strong improvement over trials because the animal is not learning the escape location. The spatial search strategy, in contrast, results in longer latencies to escape when the escape is in a novel location (i.e., trial 1) but will become shorter as the escape location is learned over subsequent trials. We assessed search strategy for each mouse in our study and found that only half of the pair housed mice (3 of 6) were using spatial search strategies to locate the escape while significantly more (13 out of 14) group housed mice used spatial search (Figure 4B). These findings suggest that group-housed mice use neural processes that rely on hippocampal function in the Barnes maze while pair-housed mice rely on non-hippocampal processes.

### Group Housing Does Not Alter Sociability or Social Memory

**Figure 5:**
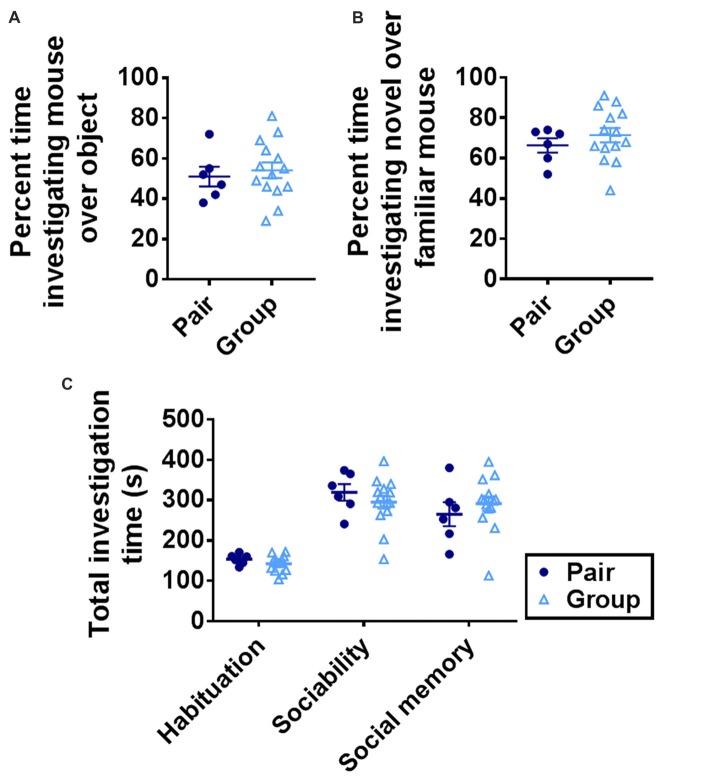
**(A)** Group- and pair-housed mice did not differ in percent time spent investigating a novel mouse over a novel object in the sociability task. Unpaired Welch’s test, *p* = 0.625. **(B)** Group- and pair-housed mice did not differ in percent time spent investigating a novel mouse over a familiar mouse in the social memory task. Unpaired Welch’s test, *p* = 0.327. **(C)** Total time spent investigating both chambers increased over habituation, sociability or social memory trials but did not differ between group- and pair-housed mice. Two-way RM-ANOVA: main effect of trial (*p* < 0.0001), main effect of housing (*p* = 0.866), trial by housing interaction (*p* = 0.221), effect of subjects (*p* = 0.008). Each point represents one mouse. Average ± SEM is shown.

Preference for interaction with a conspecific (sociability) and preference for a novel conspecific over a familiar one (social memory) can deteriorate with age (Shoji et al., 2016). These processes rely on multiple brain regions, including the basolateral amygdala and ventral hippocampus (Felix-Ortiz and Tye, 2014). To determine whether group housing affected social preference or social memory, mice were tested in a 3-chamber sociability task (Moy et al., 2004). Group-housed and pair-housed mice did not differ in investigation time of a novel mouse vs. a novel object or of a novel mouse over a familiar one (Figures 5A,B). Total investigation time increased significantly over trials but did not differ between housing conditions on any trial (Figure 5C). These findings suggest that sociability and social memory are not altered by group housing.

### Group Housing Does Not Affect Body Weight or Basal Corticosterone

**Figure 6:**
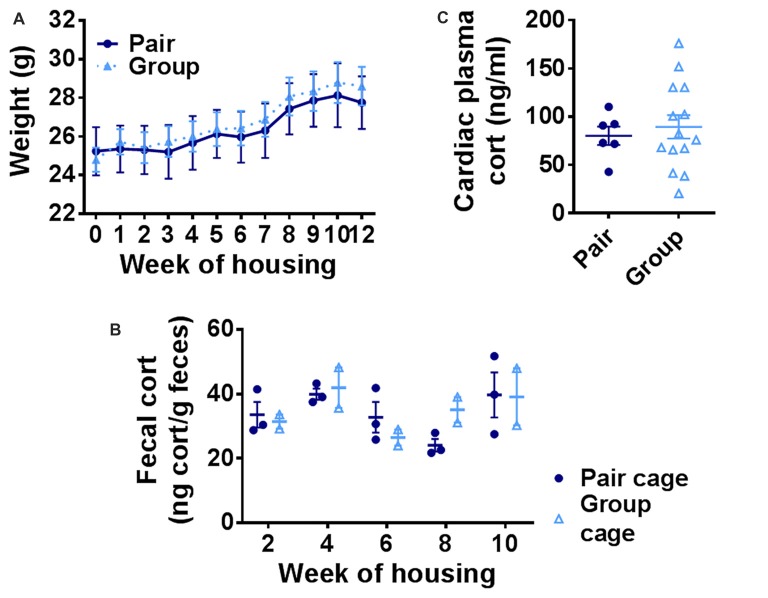
**(A)** Body weight increased over the course of housing but did not differ between group- and pair-housed mice. Two-way RM-ANOVA: main effect of week (*p* < 0.0001), main effect of housing (*p* = 0.798), trial by housing interaction (*p* = 0.840), effect of subjects (*p* < 0.0001). Each point represents average ± SEM. **(B)** Fecal corticosterone metabolite levels were similar between pair-housed cages and group-housed cages. Each point represents one cage. Average ± SEM is shown. **(C)** Plasma corticosterone did not differ between group- and pair-housed mice. Unpaired Welch’s test, *p* = 0.553. Each point represents one mouse. Average ± SEM is shown.

A prominent hypothesis of aging proposes that age-related deterioration in hippocampal function results from the cumulative effects of increasing basal stress levels (Sapolsky et al., 1986). It is also hypothesized that social network-related protection against age-related cognitive decline in humans could rely on buffering of stress. We therefore asked whether group-housed mice showed signs of reduced basal stress levels by quantifying body weight and basal stress hormone (corticosterone) levels in pair- and group-housed mice. Pair- and group-housed mice showed significant weight gain throughout the housing manipulation, but did not differ from each other (Figure 6A). Because chronic stress in rodents is typically associated with impaired weight gain (Harris, 2014), these findings suggest that neither housing condition was substantially more stressful than the other. To assess stress hormone levels more directly, we used fecal sampling to non-invasively measure basal corticosterone, the primary mouse glucocorticoid, throughout the housing period. No notable difference was found in fecal levels of corticosterone metabolites between group cages and pair cages (Figure 6B). No difference was found in individual plasma corticosterone from cardiac blood taken at euthanasia between the two housing conditions either (Figure 6C). These findings suggest that there is no gross difference in basal stress levels between pair- and group-housed mice.

### Group Housing Reduces Microgliosis in the Hippocampus

**Figure 7:**
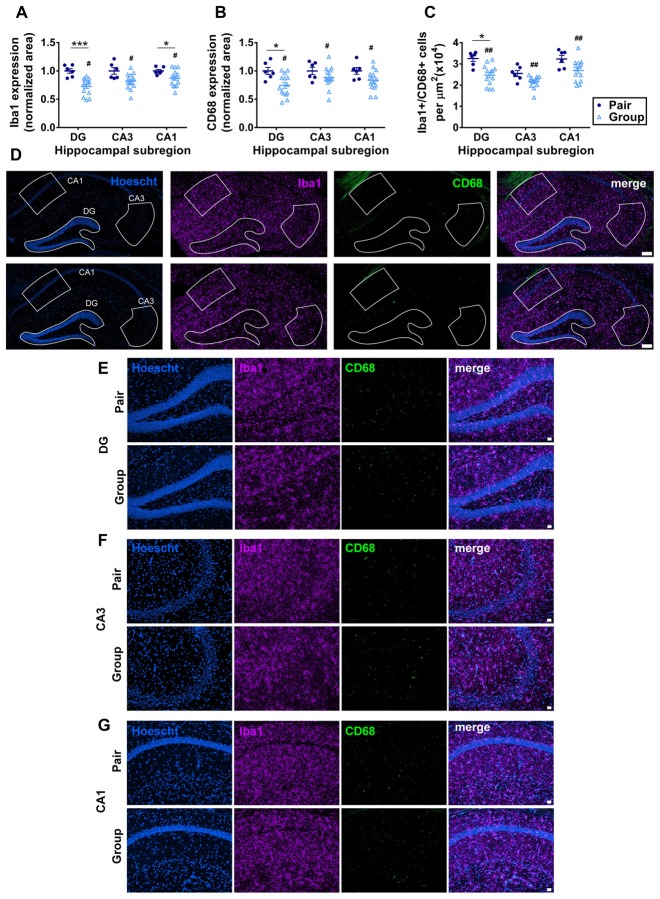
**(A)** Percent thresholded area of Iba1 immunoreactivity was significantly less in the hippocampus of group-housed over pair-housed mice, particularly in the dentate gyrus (DG). Two-way RM-ANOVA: main effect of area (*p* = 0.021), main effect of housing (^#^*p* = 0.004), area by housing interaction (*p* = 0.021), effect of subjects (*p* < 0.0001). ***p = 0.0009, *p = 0.041, *post hoc* Welch’s test with Bonferoni correction. **(B)** Percent thresholded area of CD68 immunoreactivity was significantly less in the hippocampus of group-housed over pair-housed mice, particularly in the DG. Two-way RM-ANOVA: main effect of area (*p* = 0.352), main effect of housing (^#^*p* = 0.015), area by housing interaction (*p* = 0.352), effect of subjects (*p* = 0.004). *p = 0.023 *post hoc* Welch’s test with Bonferoni correction. In both **(A,B)**, each point represents one mouse, normalized to the pair-housed average by brain area. Average ± SEM is also shown. **(C)** Number of Iba1/CD68 immunoreactive cells per μm^2^ was significantly less in the hippocampus of group-housed over pair-housed mice, particularly in the DG. Two-way RM-ANOVA: main effect of area (*p* < 0.0001), main effect of housing (^##^*p* = 0.003), area by housing interaction (*p* = 0.108). *p = 0.027 *post hoc* Welch’s test with Bonferoni correction. **(D)** Representative images of Iba1 (purple) and CD68 (green) immunoreactivity in hippocampus of pair- and group-housed mice, including representative outlines of different hippocampal subregions for quantification. Hoescht (blue) provides cell nuclei labeling. Scale bar is 100 μm. **(E–G)** Higher magnification representative images of Iba1 (purple) and CD68 (green) immunoreactivity in DG **(E)**, CA3 **(F)** and CA1 **(G)** of pair- and group-housed mice. Hoescht (blue) provides cell nuclei labeling. Scale bar is 20 μm.

Aging is commonly associated with increased inflammation both systemically and within the brain (Xia et al., 2016). In the brain, the hippocampus is particularly susceptible to signs of gliosis, an increase in inflammation of local microglia which impair hippocampal function (Barrientos et al., 2015; Pardo et al., 2017). To determine whether the improvements in cognition found in behavioral tasks were associated with underlying changes in hippocampal neuroinflammation, we quantified microgliosis in the three major subdivisions of the hippocampus: the dentate gyrus (DG), CA3 and CA1. Microgliosis was measured by immunoreactivity of Iba1 (which is expressed by all microglia) and CD68 (which is expressed by activated microglia). Iba1 immunoreactive area was significantly decreased in group-housed mice compared to pair-housed mice, most prominently in the DG and CA3 (Figures 7A,D–G). CD68 immunoreactive area was similarly reduced in group housed mice overall, with the most prominent decrease in the DG (Figures 7B,D–G). All Iba1+ cells were at least partially CD68+, consistent with an overall high level of microglial activation in these aged mice. The number of Iba1+/CD68+ microglia was similarly reduced in the hippocampus of group-housed mice relative to pair-housed mice, particularly in the DG (Figure 7C). These findings suggest reduced microgliosis in the DG of group-housed mice due at least partially to a reduction in total number of Iba1/CD68+ microglia.

### Group Housing Does Not Alter Adult Neurogenesis

**Figure 8:**
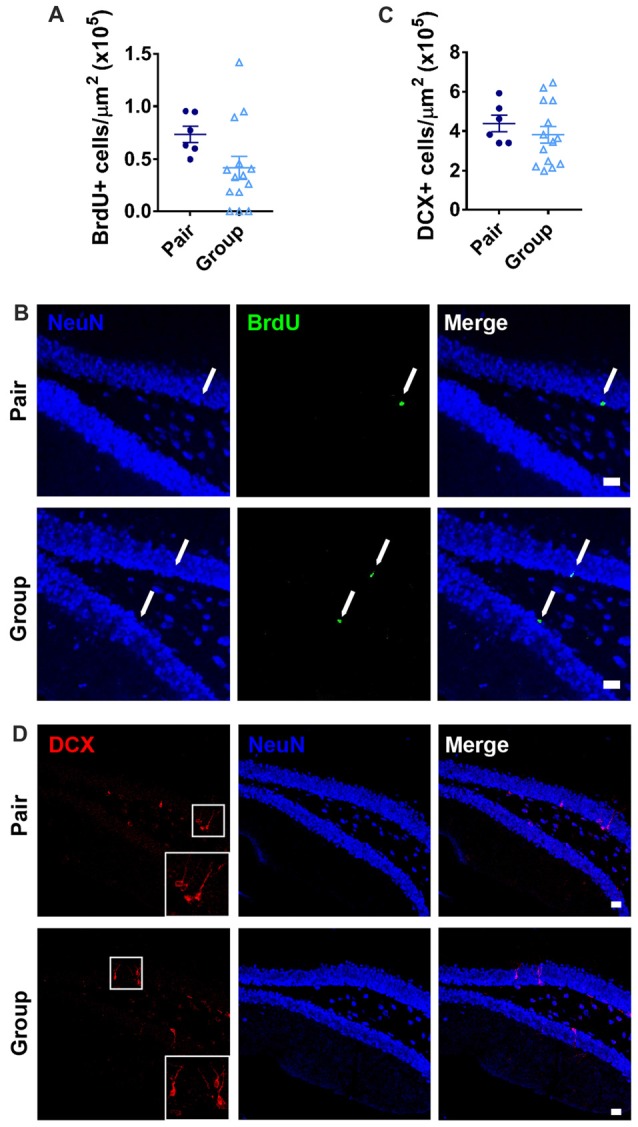
**(A)** Pair- and group-housed mice were injected with BrdU 4 weeks before perfusion. No difference in BrdU+ cell number per μm^2^ in the DG was found between housing conditions. Unpaired Welch’s test, *p* = 0.089. **(B)** Representative images of BrdU-immunoreactive cells are shown with NeuN immunoreactivity provided for anatomical orientation. Arrows point to BrdU+ cells in the subgranular zone. **(C)** Doublecortin (DCX) immunoreactivity in the DG revealed no difference in DCX+ cell number per μm^2^ between pair- and group-housed mice. Unpaired Welch’s test *p* = 0.350. **(D)** Representative images of DCX-immunoreactive cells are shown with NeuN immunoreactivity provided for anatomical orientation. White box is shown magnified in inset. In **(B,D)**, scale bars are 20 μm.

In addition to age-related neuroinflammation, the aging brain also shows a prominent decline in the birth of new neurons, or adult neurogenesis, that is associated with memory dysfunction (Lee et al., 2012). Within the hippocampus, adult neurogenesis occurs selectively in the DG where resident stem and progenitor cells proliferate and give rise to new neurons that integrate in to local circuitry (Gonçalves et al., 2016). In aging, proliferative activity of the neural stem and progenitor cells declines precipitously, resulting in a decrease in new neuron number (Lee et al., 2012; DeCarolis et al., 2015). To determine whether housing conditions affected the number of newly proliferated cells in the DG, we labeled dividing cells with the mitotic marker BrdU 1 month before perfusion. As expected for aged mice, the total number of BrdU+ cells in the DG was over all very low. BrdU+ cell number was not significantly different between group- and pair-housed mice (Figures 8A,B). There were too few BrdU+ cells to accurately assess neuronal cell fate within this population. Therefore, to better assess the number of new neurons, the number of cells expressing doublecortin (DCX), a marker of immature neurons, was quantified. Group- and pair-housed mice did not differ from each other in number of DCX+ immature new neurons in the DG (Figures 8C,D).

## Discussion

### Effect of Social Network on Age-Related Cognitive Decline

Aging is broadly associated with progressive impairment in hippocampal function in mammals (Bettio et al., 2017). Both cross-sectional and longitudinal studies suggest that humans with more social ties have better preservation of cognitive function, particularly hippocampal-dependent forms of memory, in old age than those with fewer ties (Bassuk et al., 1999; Seeman et al., 2001; Béland et al., 2005; Ertel et al., 2008; Haslam et al., 2015; Brown et al., 2016). Causation, however, has been difficult to determine in humans and clinical trials of social network interventions have yielded unclear results (Hogan et al., 2002; Green et al., 2008).

Mice, like humans, are social and spontaneously associate with conspecifics both in captivity and in the wild. We found that housing mice in larger groups led to improved novel object location memory compared to housing in pairs. Novel object location memory relies primarily on hippocampal neural processes and deteriorates prominently with age (Fahlström et al., 2011). Non-hippocampus-dependent novel object recognition, in contrast, was well-preserved in both pair- and group-housed mice (Bachevalier and Nemanic, 2007; Komorowski et al., 2009; Barker and Warburton, 2011; Fahlström et al., 2011). In the Barnes maze, a spatial navigation task that also shows age-related impairment, we did not find a difference in overall performance between pair and group-housed mice. While this version of the Barnes maze has been shown to be sensitive to improved hippocampal function in aged mice (Castellano et al., 2017), changes in latency to escape in the present study were minimal in both groups, providing little evidence of learning. These findings suggesting the task was too difficult to master in the time allotted. However, we did find that group-housed mice showed a stronger preference for using spatial search strategies than pair-housed mice, albeit with a small sample size for detecting such an effect. Previous studies show that aging often leads to a shift from more accurate, but difficult, spatial search strategies to less accurate, but less cognitively demanding, serial strategies in both rodents and humans (Bach et al., 1999; Konishi et al., 2017). This shift is hypothesized to reflect an age-related failure of hippocampal processes necessary to support spatial search. Our findings of spatial search strategy preference in group-housed mice therefore suggests greater reliance on hippocampal processes, possibly because they are better preserved in these mice compared to pair-housed mice (Bassuk et al., 1999; Hogan et al., 2002; Green et al., 2008).

In contrast to spatial memory, social memory relies on several brain regions that are resistant to age-related decline (Felix-Ortiz and Tye, 2014). We found no differences in sociability or social memory in pair- vs. group-housed mice. However, it is possible that more subtle differences in social behavior could be found in tests that allow direct contact between animals or with longer delays for memory recall. The present work did not include measures of home cage social behavior (agonistic interactions, grooming), for example. Resident-intruder tests might also reveal more insight in to social behaviors in group- vs. pair-housed mice. Further research will be required to better understand how social network size impacts sociability in aged mice.

### Effect of Social Network on Age-Related Neuroinflammation and Neurogenesis

Aging is associated with an increase in neuroinflammation in humans and rodents (Barrientos et al., 2015; Pardo et al., 2017). This inflammation manifests in increased pro-inflammatory cytokine expression by resident microglia, as well as microglia proliferation, and is found markedly in the hippocampus (Barrientos et al., 2015; Pardo et al., 2017). Some theories of brain aging propose that neuroinflammation is an underlying cause of age-related hippocampal cognitive decline, a process that has been termed inflammaging (Franceschi and Campisi, 2014). We found that group housing led to reduced microgliosis as reflected by decreased Iba1 and CD68 immunoreactivity and decreased number of Iba1+/CD68+ cells in the hippocampus compared to pair housing, a finding which suggests that larger social network size may protect against age-related microglial proliferation and neuroinflammation.

The activity of hippocampal stem and progenitor cells declines dramatically with age and the resultant reduced adult neurogenesis has been postulated to contribute to age-related hippocampal dysfunction (Lee et al., 2012). In the present study, neurogenesis levels were very low, and no difference in adult neurogenesis between pair- and group-housed mice was found. Previous studies show that the decline in adult neurogenesis occurs relatively early in aging, with 85%–90% reductions being observed by 12 months (Bondolfi et al., 2004; Ben Abdallah et al., 2010). The present study began at 15 months of age, long after the most prominent decline in neurogenesis was complete. Future research could address whether altering social network size earlier in aging could prevent the decline in neurogenesis.

### Effect of Environmental Enrichment on Age-Related Memory Decline

Numerous studies show that environmental enrichment can protect against age-related hippocampal dysfunction in rats and mice (van Praag et al., 2000; Kempermann et al., 2002; Kobayashi et al., 2002; Frick and Fernandez, 2003; Frick et al., 2003; Bennett et al., 2006; Harburger et al., 2007; Greenwood et al., 2009; McKinnon et al., 2009; Freret et al., 2012; Speisman et al., 2013; Sampedro-Piquero et al., 2014; Bergami et al., 2015; Mora-Gallegos et al., 2015; Pérez-Martín et al., 2016). Aged animals may even be particularly susceptible to the beneficial effects of enrichment compared to younger animals (Harburger et al., 2007; Harati et al., 2011). Rodent environmental enrichment is implemented in widely varying ways across studies, often combining increased social network size with exercise (running wheel) and/or addition of a rotating cast of novel objects (i.e., toys). While the separable roles of exercise and novel objects has been investigated (van Praag et al., 1999; Fabel et al., 2009), the role of social interactions as a single variable has received relatively little attention. Some studies have even concluded that social interactions have minimal impact on hippocampal function in young animals (Rosenzweig et al., 1978; Pietropaolo et al., 2004; Madroñal et al., 2010; Brenes et al., 2015). However, aging animals may be particularly vulnerable to the effects of social interactions. For example, a study of a transgenic mouse model of Alzheimer’s-like pathology showed that co-housing with a wildtype cage mate improved memory function over housing with another transgenic mouse (Hsiao et al., 2014). These findings suggest a potential greater vulnerability of aged animals to the deleterious effects of an impoverished social environment.

### Hypotheses of Social Network Impact on Cognitive Aging

Several mechanistic hypotheses have been proposed to explain the connection between social network size and age-related cognitive decline in humans. Some researchers have proposed that larger social networks enable better cognitive aging through instrumental support that improves physical health, such as increased access to medical advice, physical support in daily functions, or access to community resources. The present study used a social rodent model where physical environment and care were controlled. Our results therefore suggest that social network size can drive improvements in memory function independent of instrumental support.

Having more numerous social ties has also been proposed to protect against cognitive decline in aging via improved emotional support and stress buffering (Ozbay et al., 2008; Sherman et al., 2016). Long-term exposure to elevated glucocorticoid hormone levels is associated with hippocampal dysfunction and neuroinflammation (Sapolsky et al., 1986; Lupien et al., 1998; Yau and Seckl, 2012; Barrientos et al., 2015). We found no differences in markers of chronic stress in group- vs. pair-housed mice. Body weight, fecal corticosterone and plasma corticosterone were all similar between the two housing groups. Nonetheless, it is still possible that housing conditions could alter response to a stressor. Over-grooming was found in two of the pair-housed mice, resulting in their and their cage mates’ elimination from the experiment. The meaning of over-grooming behavior is debated but it could be related to stress or dominance hierarchies (Militzer and Wecker, 1986; Garner et al., 2004a). Over-grooming is estimated to occur in approximately 7.7% percent of laboratory-housed mice, with risk factors for higher rates of over-grooming including older age, being female and being C57/Bl6 strain (Garner et al., 2004b). Given these risk factors, the observed frequency of over-grooming in the present study appears quite low. However, it is still possible that the presence of over-grooming in pair housing conditions is an indicator of greater stress in these mice, though the sample size of the current study was not powered to detect such low frequency events. Future research should more specifically test stress response in group- vs. pair-housed mice.

A third hypothesis of social network modulation of cognitive aging suggests that the daily interaction and cognitive load dictated by larger social networks is stimulatory itself and provides an intense enrichment that preserves brain function in old age. In support of this hypothesis, two recent studies in humans show that ties to a social group are more predictive of preserved cognition in aging humans than ties to a series of individual (i.e., pair-based) ties (Haslam et al., 2014, 2015). These studies suggest that the complexity of group interactions may provide a level of stimulation or cognitive demand that is protective for the aging brain. Our findings provide potential support for this cognitive enrichment hypothesis. The cognitive load represented by gauging interactions, judging hierarchy and making social decisions as one node in a seven node network is likely much greater than that presented by a single pair relationship. Further experiments are needed to confirm the benefit of group interactions over pair-wise interactions with a similar total number of mice. For example, one could compare the memory benefits of daily exposure to six other mice in isolated pair interactions vs. exposure to six other conspecifics simultaneously in shared housing.

### Limitations of the Present Work

The present study compared housing in pairs vs. housing in groups using the same physical environment as a model for having large vs. small numbers of social ties. However, there are possible cofounding differences other than number of social ties in a cage that could have driven the observed effects. Interactions and activity in the home cages were not monitored systematically. It is, therefore, possible that mice living with more cage mates simply move more, resulting in more exercise. Exercise is clearly linked to improved hippocampal function in aged mice. However, exercise also reduces body weight in aged mice and increases adult neurogenesis across the lifespan (Wu et al., 2008; Lee et al., 2012). We saw no evidence of differences in body weight or adult neurogenesis between pair and group-housed mice, suggesting that large differences in activity level in the home cages were not likely present. However, future studies could address this more specifically with home cage observations.

Another possible difference between pair- and group-housed mice stems from the size of the home cage. We used the same size home cage for pairs and for groups, resulting in smaller cage volume per mouse in the group-housed mice than in the pair-housed mice. We chose to use the same size cage for both conditions because larger physical environments are likely enriching (Bernstein, 1973), meaning that a larger cage for the larger group size would create a confounding variable of greater physical space to explore. However, it is possible some kind of thermoregulatory benefit from a greater number of bodies per cm^3^ could have played a role in the preservation of hippocampal function that we observed.

The present study was limited to female animals to avoid the high aggression levels males might display. Future studies in males conducted with extra care taken to reduce aggression could be useful. Male and females may show different effects of social housing on hippocampal physiology (Tzeng et al., 2017) and how these sex differences may interact with aging memory functions warrants further exploration.

Future studies could greatly enhance these findings by including multiple group sizes, ranging from isolated, single-housed to larger groups. It remains undetermined whether number of cage mates enhances hippocampal function dose-dependently or whether there is a plateau level beyond which no additional benefit can be derived. The present work also does not reveal how different numbers of cage mates compare to complete social deprivation. Future work could also compare more directly how the change in social network size, as opposed to total size, affects hippocampal function. The mice in this experiment were housed four per cage in an NIA colony and in-house before experimental manipulation began, resulting in a decrease in social network size for pair-housed mice and an increase for group-housed mice. To better mimic human aging trajectories, future work could start with mice housed in large groups at a much younger age then reduce cage mate number as they age to determine how changes in network size over time affect hippocampal health. Such a study would also address another confound of the present study: by necessity, some of the group-housed mice were familiar to each other before the housing manipulation began, whereas pair-housed mice were not. The number of familiar mice was kept minimal (2 mice per cage of 7), but it is still possible that this familiarity between two mice influenced the entire group’s response to housing. A long-term aging study where cage mates are removed over time would address this difficulty by using only mice familiar to each other throughout.

All of the animals in the present study underwent behavioral testing before perfusion. Behavioral testing itself can alter brain function, including markers of hippocampal integrity (Sampedro-Piquero et al., 2014). It is therefore possible that the observed differences in hippocampal neuroinflammation rely on the presence of behavioral testing to emerge. Future studies can pursue replication of these effects in testing-naïve mice. However, the presence of behavioral testing is not entirely inappropriate, given that aging humans live in complex environments that tax memory on a daily basis. Therefore, any effect of social ties on human hippocampal function likely occur on a background on daily cognitive tasks.

### Summary

As medical care improvements lengthen the average lifespan, preservation of quality of life is becoming a more urgent public health goal. Cognitive decline can dramatically impair function and wellness in aged individuals. However, there is great heterogeneity in decline, with some individuals appearing quite resilient (Negash et al., 2011, 2013). Common correlates of resilience present potential avenues of treatment or prevention if those correlates are indeed causative. Current non-pharmacological approaches to preserving memory function in old age include dietary restriction, physical exercise, cognitive stimulation and stress management, among others (Bettio et al., 2017). Our findings in a mouse model of social network manipulation during aging suggest that encouraging participation in larger social networks may also be a viable non-pharmacological treatment for age-related memory decline. Notably, other methods of preventing cognitive decline, such as dietary restriction and exercise, often suffer from poor adherence rates (Anton and Leeuwenburgh, 2013). In contrast, a recent survey found that elderly individuals will combat numerous obstacles (poor health, pain, hazardous conditions) to achieve social engagement (Gardner, 2013). Encouraging social participation and establishing communities conducive to maintained social engagement, such as co-housing communities (Williams, 2005), may therefore be a particularly practical method for prevention or mitigation of cognitive decline.

## Author Contributions

BS, XY and KC performed experiments, analyzed data and edited/reviewed the manuscript. EK performed and designed experiments, analyzed data and wrote the manuscript.

## Conflict of Interest Statement

The authors declare that the research was conducted in the absence of any commercial or financial relationships that could be construed as a potential conflict of interest.

## Supplementary Material

FIGURE S1 Adult C57/Bl6 mice were fed PLX5622 or vehicle diet for 21 days. PLX5622 diet led to an almost complete loss of Iba1+ and CD68+ immunoreactivity, shown here in representative images at lower **(A)** and higher magnifications **(B)**. An example Iba1+/CD68+ cell from a vehicle diet treated mouse is also shown **(C)**. Scale bars are **(A)** 200 **(B)** 50 and **(C)** 10 μm.
