# Supplementary figures and images for: A Larger Social Network Enhances Novel Object Location Memory and Reduces Hippocampal Microgliosis in Aged Mice
Source: Front Aging Neurosci. 2018 May 31;10:142. doi: 10.3389/fnagi.2018.00142 (PMC5990613; doi:10.3389/fnagi.2018.00142)

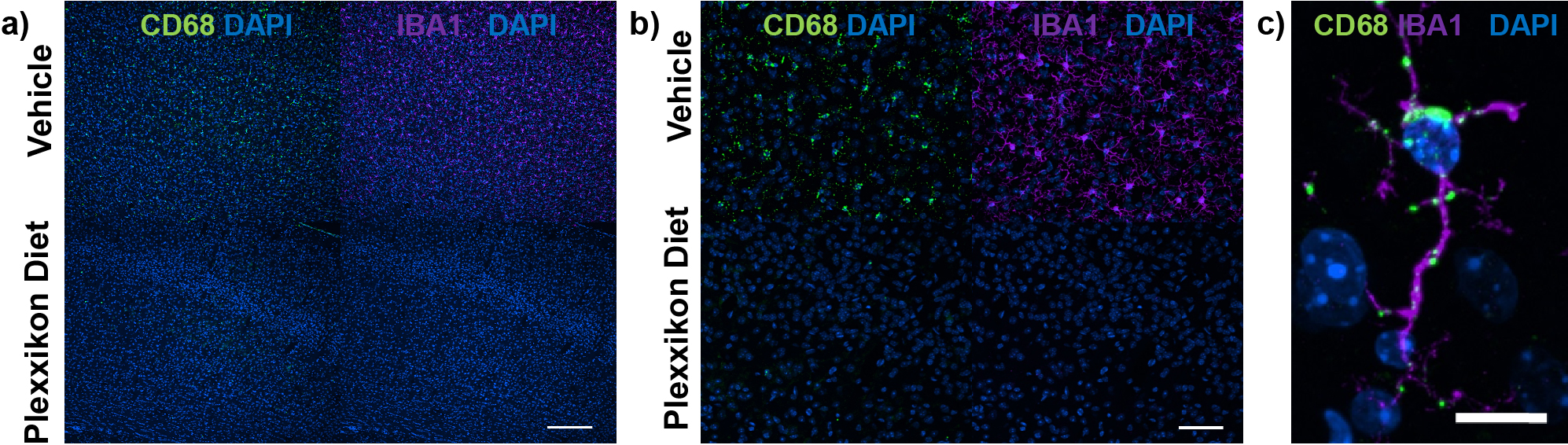

Supplement: FIGURE S1 — Adult C57/Bl6 mice were fed PLX5622 or vehicle diet for 21 days. PLX5622 diet led to an almost complete loss of Iba1+ and CD68+ immunoreactivity, shown here in representative images at lower (A) and higher magnifications (B). An example Iba1+/CD68+ cell from a vehicle diet treated mouse is also shown (C). Scale bars are (A) 200 (B) 50 and (C) 10 μm. [file Image_1.JPEG]
